# Supplementary material for: Picosecond-scale heterogeneous melting of metals at extreme non-equilibrium states
Source: Nat Commun. 2025 Nov 25;16:10464. doi: 10.1038/s41467-025-65485-6 (PMC12647707; doi:10.1038/s41467-025-65485-6)
Supplement: Supplementary file 1 — Supplementary Information [file 41467_2025_65485_MOESM1_ESM.pdf]

Supplemental Information: Picosecond-scale Heterogeneous Melting of Metals At Extreme Nonequilibrium States

Qiyu Zeng,[1,2,3] Xiaoxiang Yu,[1,2,3,*] Bo Chen,[1,2,3] Shen Zhang,[1,2,3]
Kaiguo Chen,[1,2,3] Dongdong Kang,[1,2,3,†] and Jiayu Dai[1,2,3,‡]

[1] *College of Science, National University of Defense Technology, 410073 Changsha, Hunan, China*
[2] *Hunan Key Laboratory of Extreme Matter and Applications, National University of Defense Technology, 410073 Changsha, Hunan, China*
[3] *Hunan Research Center of the Basic Discipline for Physical States, National University of Defense Technology, Changsha 410073, China*

(Dated: September 9, 2025)

A. phonon spectra of W under equilibrium and non-equilibrium condition

To validate the accuracy of the ETD-NN model, lattice dynamics that need high-order derivatives of PES were investigated. We use finite displacement method to calculate the phonon dispersion with ALAMODE package [1] as a postprocessing code. The forces are calculated in $5 \times 5 \times 5$ supercell with cell lattice parameter $a_0 = 3.17104$ Å. The atomic displacement is set to 0.01 Å, and the interatomic force constants are extracted from KS-DFT and DPMD calculation respectively. The dynamical matrices are derived from these force displacement data to obtain phonon dispersion spectra.

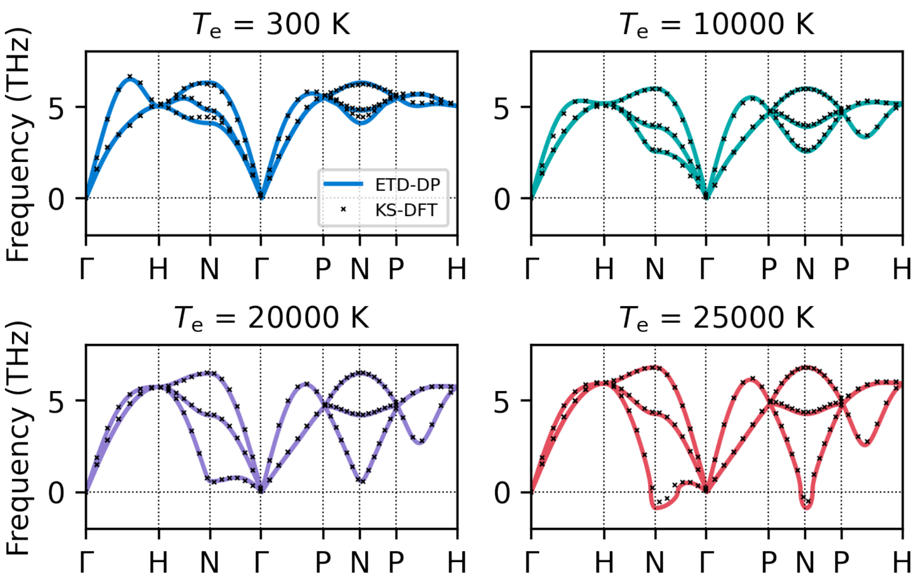

Supplementary Figure 1: Phonon spectra of two-temperature tungsten ($\rho_0 = 19.15$ g/cm^3), colored solid line indicates the DP calculation, while the black cross indicates the results obtained through KS-DFT calculations.

From Supplementary Fig.1 we can see, phonon spectra predicted by neural network model show good agreement with individual calculations by using Kohn-Sham DFT method, under both equilibrium condition and non-equilibrium condition. Moreover, the lattice stability of laser-excited tungsten is discussed in detail. At elevated electron temperature, phonon modes with a lower frequency are observed along the H − N and N − Γ path in the first Brillouin zone. This directional phonon softening behavior can be attributed to half-filled *d* bands in this transition metal. The

* xxyu@nudt.edu.cn
† ddkang@nudt.edu.cn
‡ jydai@nudt.edu.cn

depopulation of such a strong directional component in electronic bonding weakens the directional forces and may drive the crystalline structure towards close-packed forms.

By further increasing the electron temperature, the imaginary phonon frequency at N-point is observed, indicating that the initial BCC structure is dynamically stable under isochoric condition at $T_e \geq 22000$ K. In the main text, we mainly focus on the moderate non-equilibrium condition below the $T_e = 20,000$ K to exclude the influence of lattice-instability-driven nonthermal phase transition.

B. Thermodynamic profile and structural transformation of laser-excited W nanofilm

For comparison, we performed TTM-DPMD simulations with ground-state PES $A(\mathcal{R}, T_\mathrm{i})$ to obtain the temporal evolution of thermodynamic profile, as shown in Supplementary Fig.2(a). The laser fluence is set to 120 mJ cm^{-2}. During this purely thermal process, the dynamic process is dominated by the electron-phonon coupling. As presented in the main text, the evolution stages can be divided into three part: (1) isochoric heating of lattice through electron-phonon coupling, (2) uniaxial expansion in response to the thermal kinetic pressure of heated ions, which is not obvious compared with that under nonthermal condition, (3) thermal melting as lattice temperature increases above the limit of lattice thermal stability, accompanied by absorption of latent heat.

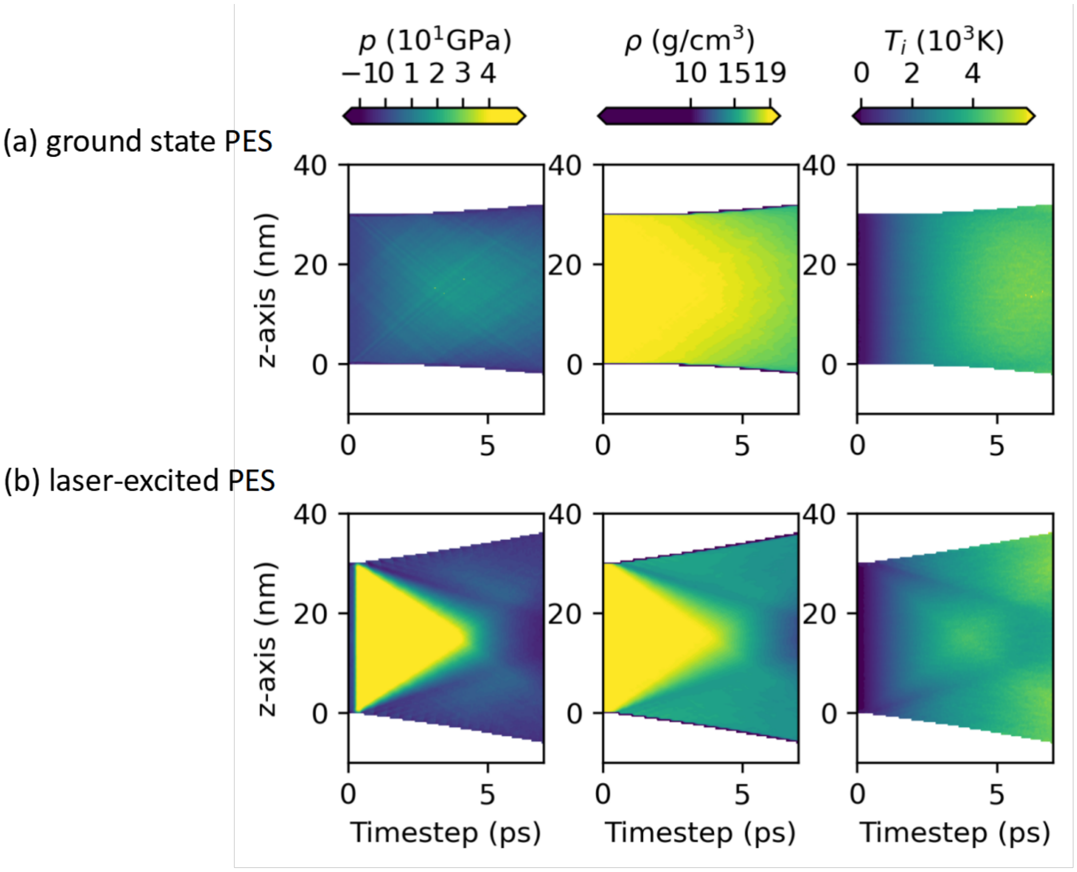

Supplementary Figure 2: Thermodynamic profile (pressure p, density ρ, and lattice temperature T_i) of laser-excited tungsten nanofilm (a) during a purely thermal process (b) nonthermal process, where absorbed laser fluence is set to 120 mJ cm^{-2}.

As shown in Supplementary Fig.2(a), the lattice is heated via electron-phonon energy exchange and thermal kinetic pressure is accumulated. During purely thermal process, the surface velocity increases gradually in response to thermal kinetic pressure, and reaches its maximum value of ~ 640 m s^{-1} at t = 8 ps (see Supplementary Fig.3(a)). However, such expansion plays negligible role in creating density gradient and whole sample experiences homogeneous density decrease of ~ 2 g/cm^3.

Once the hot-electron-contributed pressure be included by using laser-excited PES $A(\mathcal{R}, T_\mathrm{e})$, a superhigh surface velocity of ~ 755 m s^{-1} is obtained at the initial stage and keeps increasing up to ~ 1070 m s^{-1}. With such

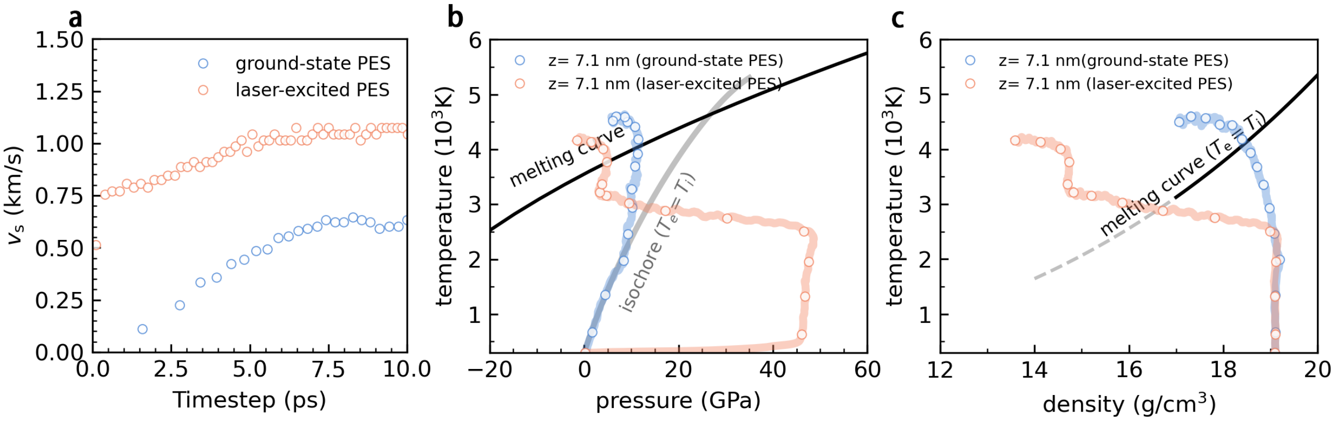

Supplementary Figure 3: (a) The temporal evolution of surface velocity (b)(c) thermodynamic pathway of selected region in laser-excited W nanofilm under laser fluence of 120 mJ cm^{-2} in the first 7 ps, where simulation results with ground-state PES are presented for comparison.

extremely high velocity, the surface region quickly expands to release the internal stress waves and lattice structure can be distorted with uniaxial strain up to $\sim 35.43\%$, corresponding density decrease to 14.13 g cm^{-3} as presented in the main text. As a result, a dramatic density decrease of around 5 g/cm^3 is observed (see Supplementary Fig.3(c)).

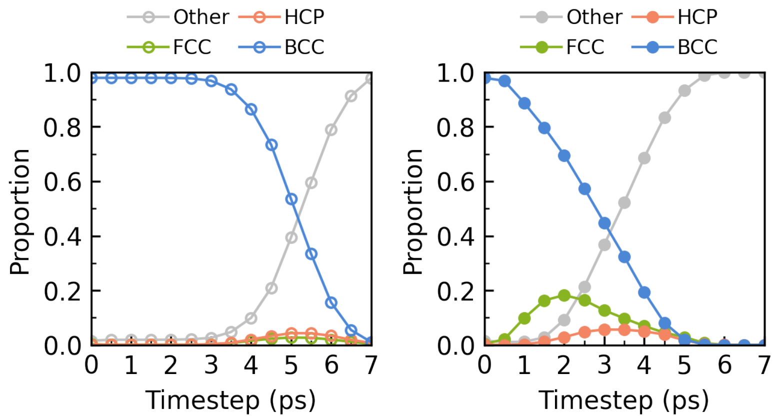

Supplementary Figure 4: Ratio of different structure type during laser heating process with laser fluence of 120 mJ cm^{-2}, identified by the polyhedral template matching method [2]. The left/right panel denotes results exclude/include laser-excited PES in TTM-DPMD simulations.

Supplementary Fig.4 shows the proportion of atoms with different local environment during the laser heating process, we demonstrate that a maximum proportion around 20% of all atoms undergoes BCC-FCC phase transition as the release of nonthermal stress waves (right panel in Supplementary Fig.4). Then the concentration of FCC-coordinated atoms begins to decrease, accompanied with sharp increase of disordered structure due to the onset of ultrafast melting. As compared with traditional results (left panel in Supplementary Fig.4), these results indicates that the occurrence of the solid-solid phase transformation originates from nonthermal nature. And the lifetime of the new solid phase is determined by the interplay between electron-phonon coupling and nonthermal stress waves relaxation.

We also note that, due to the characteristics of the PTM method, a small fraction of atoms are identified as HCP during the structural evolution. These HCP-type atoms mainly arise from thermally activated defects or local distortions in the BCC region, as well as from partial atomic slips within FCC domains. However, these HCP-identified atoms are distributed sparsely and do not form any significant bulk regions. Consequently, they do not contribute to long-range structural measurements such as X-ray diffraction. Therefore, the overall phase transition process is dominated by the BCC-FCC transformation and subsequent melting.

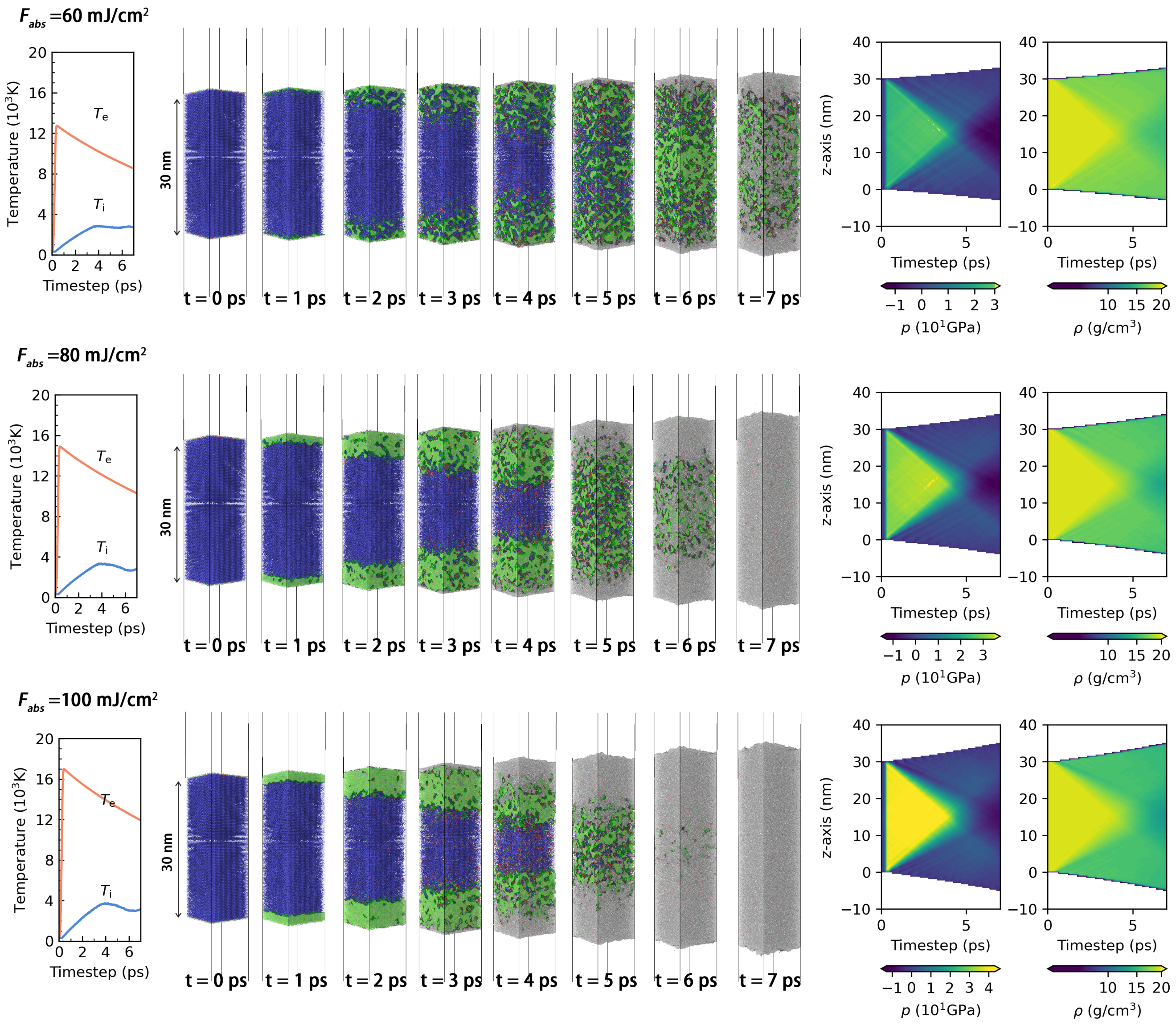

Supplementary Figure 5: Temporal evolution of electron temperature T_e, ion temperature T_i, atomic structure, pressure, and density profile of laser-excited tungsten under laser fluence of 60 mJ cm^{-2}, 80 mJ cm^{-2}, 100 mJ cm^{-2} respectively. The atomic configurations are visualized by OVITO software [3].

C. Structural transformation dynamics of laser-excited W under different laser fluence

We performed TTM-DPMD simulations across wide laser fluences, ranges from 60 mJ cm^{-2} to 100 mJ cm^{-2}. The corresponding maximum electron temperature ranges from 12770 K to 17040 K. Under such moderate non-equilibrium state, the phonon spectra indicate that the initial BCC structure is dynamically stable. As presented in the Supplementary Fig.5, under all laser fluence condition, the nonthermal pressure is built up homogeneously and the surface expansion completes at $\sim$ 4 ps. During these processes, the thermodynamic profiles exhibit obvious heterogeneous characteristics.

Under relatively low laser fluence of 60 mJ cm^{-2}, although the uniaxial expansion processes are observed, the resulted uniaxial strain is not enough to trigger solid-solid structural transformation. The maintained initial BCC symmetry is confirmed from the fragmentary characteristics of the constructed polyhedral surface meshes for FCC-type atoms. Subsequent melting behavior under such laser fluence is also discussed. The melting point of anisotropic distorted BCC structure is reduced to $\sim$ 2500 K, requiring relatively longer time ($\sim$ 5 ps) to heat up, which is comparable to the time scales of completion of pressure release. Therefore, the onset of surface melting and completion

of pressure release occurs simultaneously. Under such condition, the density distribution and melting behavior of system is not inhomogeneous any more, the superheating of lattice results in homogeneous melting process.

Under intermediate laser fluence of 80 mJ cm^{-2}, consistent with the Fig.3a in the main text, the release of non-thermal pressure $\sim$ 30 GPa is enough to trigger the BCC-FCC structural transformation. As a result, a considerable fraction of FCC-coordinated atoms can be observed. The melting point of surface region is reduced to 1900 ± 100 K, which takes $\sim$ 2 ps to reach such temperature. Therefore, the surface melting occurs and propagates inward quickly. However, since the density decrease and temperature increase of interior part is faster than the propagation of FCC-liquid melt front. At $t = 6$ ps, the homogeneous melting occurs. Under such condition, we conclude a competition between homogeneous melting and ultrafast heterogeneous melting mechanism.

Under relatively high laser fluence of 100 mJ cm^{-2}, similar to that condition presented in Fig.1b in the main text. The ultrafast heterogeneous melting behavior is clearly observed. During this process, the FCC-liquid transformation front follows the BCC-FCC transformation front, with a superhigh propagation speed of $\sim$ 2500 m s^{-1}.

D. Ultrafast electron diffraction pattern calculation

To identify the melting dynamics and determine the complete melting time, we performed the ultrafast electron diffraction simulations with DIFFRACTION package [4]. Simulated 3.2MeV electron radiation ($\lambda \sim 0.34$ pm) is used to create selected area electron diffraction (SAED) patterns. In this study, the SAED patterns aligned on the [100] axis are constructed by selecting reciprocal lattice points intersecting a 0.01Å^{-1} thick Ewald sphere slice.

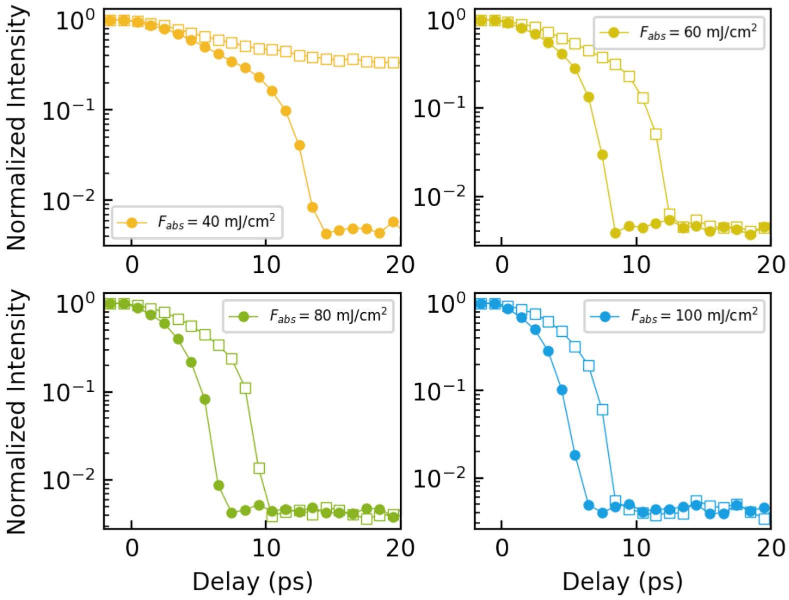

Supplementary Figure 6: Evolution of normalized intensity of (110) peak of SAED pattern, the square indicates results from ground-state PES while circles denotes results from laser-excited PES.

As shown in Supplementary Fig.6, the decay of normalized intensity of (110) peak characterizes the loss of long-range order of crystalline. Upon laser excitation, the increase of ionic temperature can result in the decrease of diffraction peaks due to the Debye-Waller effect. Under different laser fluence condition, the heights of (110) peaks relative to the adjacent background show obvious drops. As the normalized intensity decreases below 10^{-2}, accompanied with the complete disappearance of high-order diffraction peak and appearance of liquid diffraction ring, the sample is considered as completely molten.

As shown in Supplementary Fig.7, we compared the complete melting time predictions from ground-state PES and laser-excited PES. In considering of nonthermal nature, the threshold fluences to the different melting regimes are reduced, both heterogeneous and homogeneous melting are predicted to occur more rapidly.

For the purely thermal process, we can see there are only two conventional melting mechanism. At low fluence, the energy deposition by laser pulse is not enough to melt the system. At intermediate laser fluence of 40 mJ cm^{-2}, the thermal heterogeneous melting starts from the free surface and proceeds slowly by the subsonic melt-front propagation

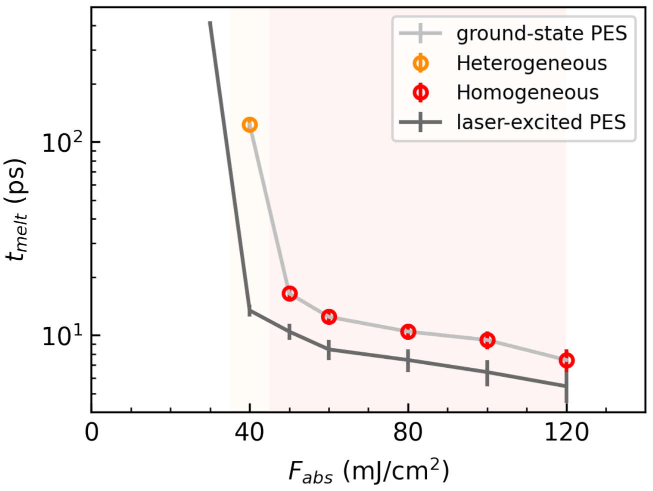

Supplementary Figure 7: Complete melting time under different laser fluence. Different colored region marks different mechanism under laser heating obtained from ground-state PES. The yellow, red region denotes the heterogeneous melting, the homogeneous melting. The dark gray line denotes the results from laser-excited PES for comparison. The error bar for the complete melting time is defined as the temporal resolution of the atomic trajectory output from the TTM-MD simulations.

(~ 125 m s^{-1}), such process lasts hundreds of picoseconds ($t_{\mathrm{m}} \sim 123 \pm 10$ ps). At high laser fluence, the homogeneous melting dominates, where the nucleation and growth of liquid region inside the foil quickly completes within several picoseconds.

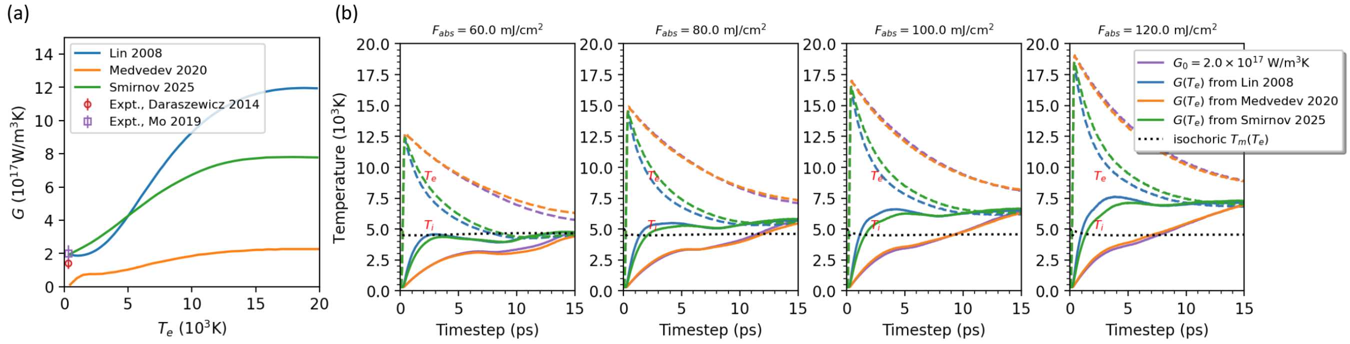

Supplementary Figure 8: Influence of $G(T_e)$ on electron-phonon energy relaxation. (a) Electron-temperature dependence of electron-phonon coupling strength, where circles/squares denote experimental measurements [5, 6], and solid lines represent theoretical predictions [7–9]. (b) Temporal evolution of electron temperature T_e (dashed lines) and ion temperature T_i (solid lines) obtained by TTM-DPMD simulations by adopting different $G(T_e)$. Black dotted line indicates the isochoric melting point of laser-excited tungsten.

E. Influence of electron-phonon coupling strength

Supplementary Fig.8(a) summarizes experimental and theoretical $G(T_e)$ values. Experimental measurements at room temperature yield $G_0 = (2.0 \pm 0.5) \times 10^{17}$ Wm^{-3}K^{-1} (UED experimental measurement by Mo *et al.* [6]) and $G_0 = (1.4 \pm 0.3) \times 10^{17}$ Wm^{-3}K^{-1} (optical pump-probe measurements by Daraszewicz *et al.* [5]). Theoretical predictions diverge significantly at high electron temperatures: at $T_e = 20,000$ K, Lin *et al.* and Smirnov *et al.* predict $\sim 12.0 \times 10^{17}$ Wm^{-3}K^{-1} [7] and $\sim 7.0 \times 10^{17}$ Wm^{-3}K^{-1} [9], respectively, while Medvedev *et al.* suggest a relatively lower value $\sim 2.0 \times 10^{17}$ Wm^{-3}K^{-1} [8].

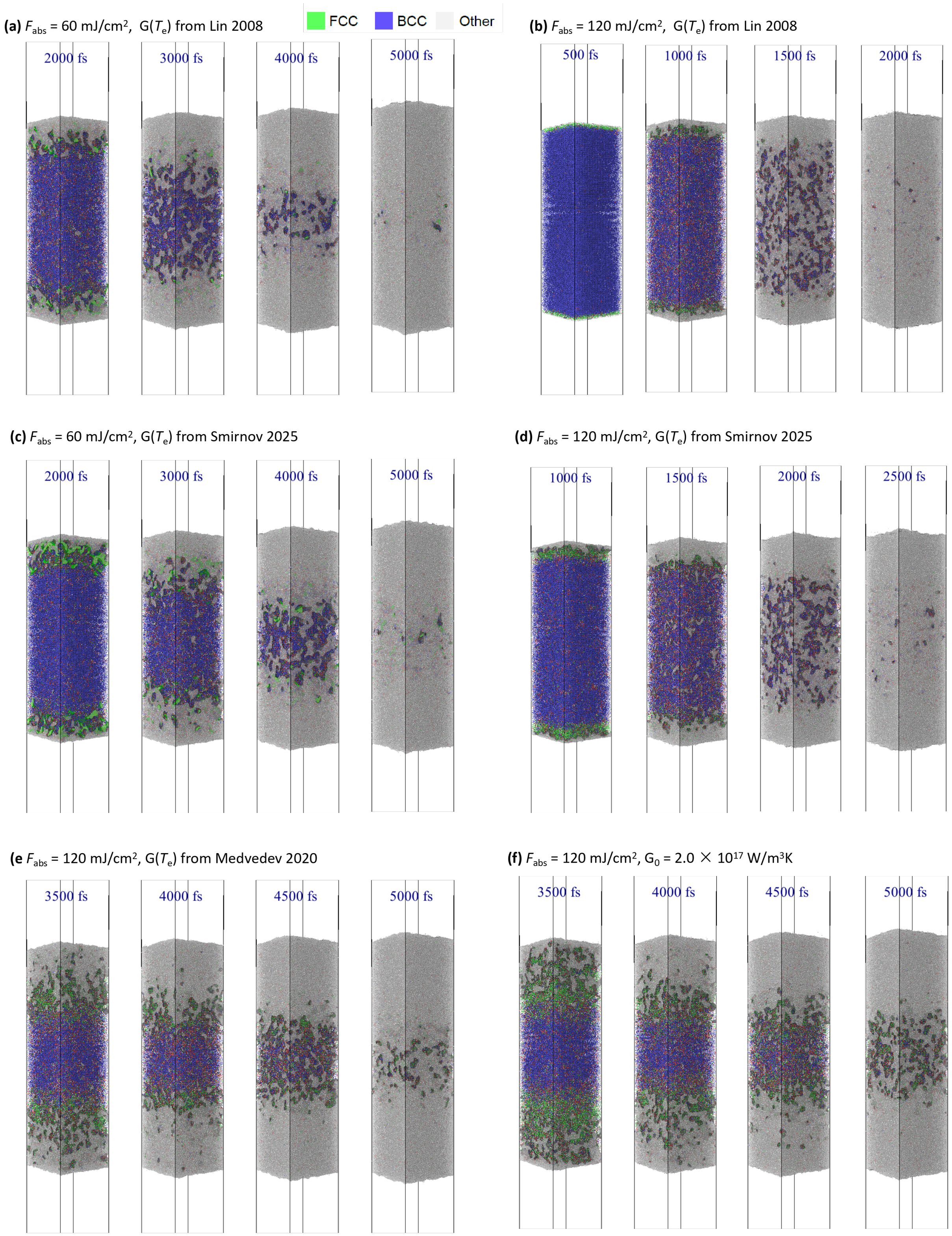

Supplementary Figure 9: Influence of $G(T_e)$ on melting dynamics. (a-f) temporal evolution of atomic configurations. Red dashed lines mark the melt fronts. Liquid local structure (gray atoms) were determined using PTM method. The atomic configurations are visualized by OVITO software [3].

Influence of $G(T_e)$ on electron-phonon energy relaxation. Supplementary Fig.8(b) shows temperature evolution from TTM-DPMD simulations. Stronger coupling models ($G(T_e)$ from Lin *et al.* or Smirnov *et al.*) induce rapid lattice heating, surpassing the isochoric melting point $T_{m,\rho_0}(T_e)$ within 3-5 ps. In contrast, a weaker coupling model ($G(T_e)$ taken from Medvedev *et al.*) exhibits heating rates comparable to constant electron-phonon coupling approximations (G_0 from Mo *et al.*'s measurement) in the main text. This results in slower thermalization where the lattice temperature exceeds the isochoric melting point within 15 ps at $F_{abs} = 60$ mJ/cm^2 and 7 ps at $F_{abs} = 120$ mJ/cm^2 respectively.

Influence of $G(T_e)$ on melting dynamics. Supplementary Fig.9 illustrates the temporal evolution of atomic configurations. In Supplementary Fig.9(a)(b), when using stronger $G(T_e)$ model from Lin *et al.*'s work, moderate fluence (60 mJ cm^{-2}) provides a comparable timescales of heating and pressure relaxation, enabling ultrafast heterogeneous melting. Above 100 mJ cm^{-2}, accelerated lattice heating induces homogeneous nucleation of liquid phase in bulk regions, completing melting prior to stress wave propagation, indicating a homogeneous melting mechanism. For $G(T_e)$ from Smirnov *et al.*, the behavior is qualitatively similar to Lin *et al.*'s $G(T_e)$ (Supplementary Fig.9(c)(d)).

Medvedev *et al.*'s weaker $G(T_e)$ reveals similar behavior to the constant G_0 case presented in the main text, see Supplementary Fig.9(e)(f). Melting initiates and completes below the isochoric melting threshold $T_i < T_{m,\rho_0}(T_e)$ across all laser fluences, indicating a dominant role of electron pressure relaxation in driving melting process. At higher fluences (> 100 mJ cm^{-2}), surface density decrease combined with rapid heating drives surface-initiated ultrafast heterogeneous melting.

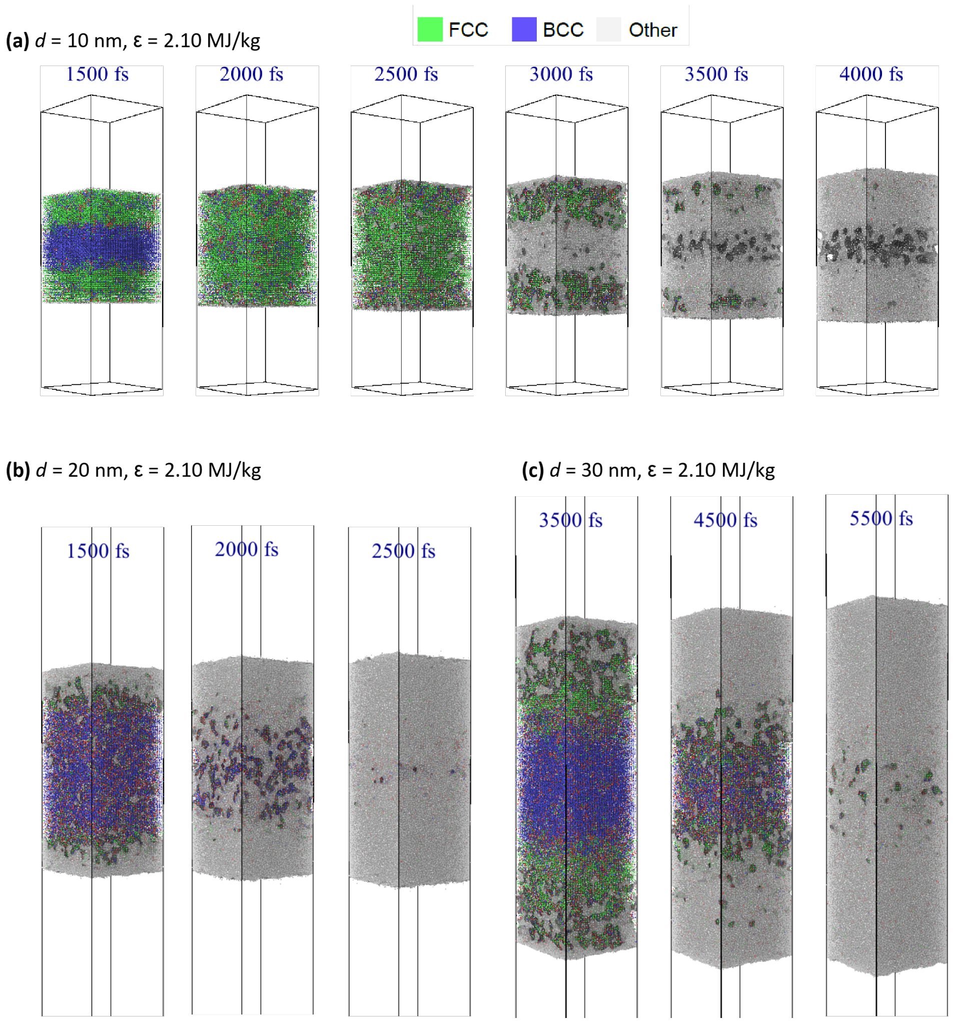

Supplementary Figure 10: Influence of sample thickness on melting dynamics. (a-c) Spatio-temporal evolution of atomic configurations under laser energy density of 2.10 MJ/kg. Red dashed lines mark the melt fronts. Local liquid structure N_{liq} were determined using PTM method. The atomic configurations are visualized by OVITO software [3].

F. Influence of sample thickness

The ultrafast heterogeneous melting process is intrinsically related to the propagation of electronic stress waves, making the film thickness d a critical factor in understanding structural transformation dynamics. Given tungsten's electron mean free path of ~ 33 nm, we conducted supplementary simulations for 10 nm and 20 nm thick W films to investigate thickness effects under uniform energy deposition. All TTM-DPMD parameters remained unchanged as compared with the 30 nm case in the main text. For consistent analysis across different geometries, we normalized the laser fluence F_{abs} to thickness-independent laser energy density ϵ through $\epsilon = F_{abs}/\rho d$.

Since the propagation velocity v of electronic stress waves is thickness-independent, the propagation time $\tau = d/v$ reduces for thinner films. Taken laser energy density of 2.10 MJ/kg as example, for the 30 nm system, electronic stress waves require approximately 4.6 ps to propagate from surface $z = 0$ nm to interior $z = d/2 = 15$ nm, while the 10 nm system completes this process within 1.5 ps. In 10 nm films (Supplementary Fig.10(a)), this reduced timescale makes electron-phonon energy exchange insufficient to reach melting condition before the stress waves converge. When the counter-propagating stress waves cross each other at 2 ps, the accumulated uniaxial distortion leads to complete BCC-FCC transformation. Subsequent outward propagation of stress waves induces further density decrease, ultimately initiating heterogeneous melting that propagates outward at ~ 3000 m/s.

While in 20 nm system (Supplementary Fig.10(b)), the characteristic time for dual surface-initiated stress waves to converge at the geometric center (~ 3 ps) becomes comparable to the thermalization timescale (2.5 ps) required to exceed the isobaric melting point. As a result, the system exhibits features of both ultrafast heterogeneous melting and homogeneous melting mechanism.

While in the 30 nm system, sufficient lattice heating occurs prior to stress wave arrival at the center (Supplementary Fig.10(c)). Under laser energy density of 2.10 MJ/kg, thermal activation in surface low-density regions initiates melting before convergence of stress waves, creating surface-initiated ultrafast heterogeneous melting phenomenon.

G. Nonthermal response of laser-excited gold

To demonstrate the ubiquity of electronic pressure in laser-driven processes, here we present short overview on nonthermal behavior of laser-excited gold (Au), a typical noble metal with fully occupied d orbits.

We generated an ETD-NN model for laser-excited Au, using the same procedures as those used for laser-excited W. Considering the possible uniaxial expansion and structural transformation dynamics, we explore ordered and disordered structures of Au under NVT ensemble, isotropic and anisotropic NPT ensemble across vast thermodynamic regime. Specifically, the electron temperature ranges from 300 K to 25,000 K, ion temperature ranges from 100 K to 7,000 K, and the pressure ranges from -1 GPa to 60 GPa. For the KS-DFT simulation settings, we employ the local density approximation (LDA) exchange correlation functional and psudopotential in the projector augmented-wave (PAW) formalism, including 11 valence electrons ($5d^{10}6s^1$). The kinetic energy cutoff is set to 600 eV to ensure an energy convergence of 1 meV/atom, and the sampling of Brillouin zone is chosen as 0.5 Å^{-1} .

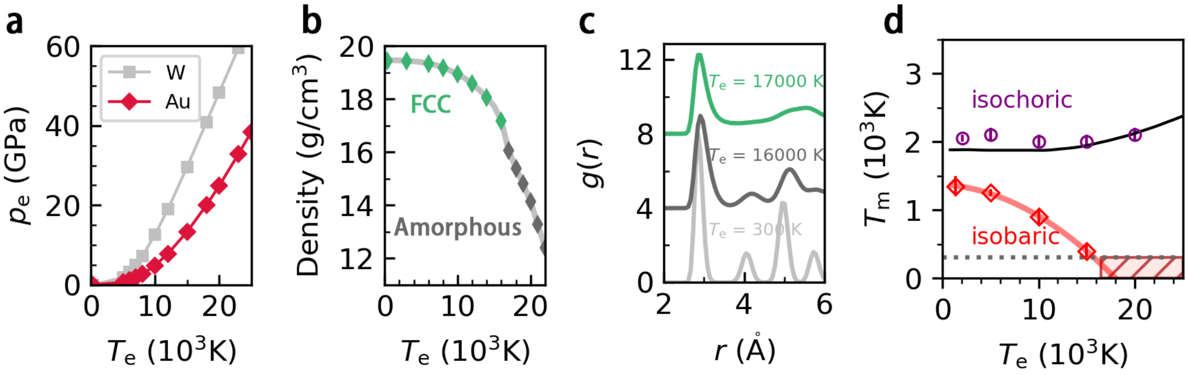

Supplementary Figure 11: Nonthermal behavior of laser-excited gold. (a) electronic pressure contributed by hot electrons. (b) density decrease and (c) short-range order of laser-excited gold under uniaxial isobaric ensemble (d) isobaric and isochoric melting behavior of laser-excited gold, the black solid line denotes previous lattice dynamics prediction by Smirnov *et al.* [10], and the nonthermal melting regime ($T_m \leq 300$ K) is filled with red diagonals. The error bar associated with the melting point is defined as half the temperature interval between the two-phase method simulations where the solid phase is stable and those where it is molten.

From Supplementary Fig.11(a), we show that the hot electrons also contribute non-negligible electronic pressure in Au, which is general in laser-excited metal systems [11]. At an electron temperature of $\sim 20,000$ K, the electron pressure can reach 25 GPa, indicating a possible relaxation dynamics if the sample surface is free.

From Supplementary Fig.11(b)(c), we discuss the structural transformation in excited Au with cold lattice (T_i = 300 K) under uniaxial NPT ensemble. As the electron temperature increases, the FCC structure transforms into face-centered tetragonal (FCT) structure due to uniaxial expansion, with corresponding decrease in density from 19.44 g cm^{-3} at $T_\mathrm{e} = 300$ K to 17.17 g cm^{-3} at $T_\mathrm{e} = 16,000$ K. As T_e reaches 17,000 K, the lattice collapses and transforms into a disordered structure with density of 16.06 g cm^{-3}. The density decrease alters the melting point, which can even drop below room temperature ($T_\mathrm{i} = 300$ K) above $T_\mathrm{e} = 17,000$ K, see Supplementary Fig.11(d). This indicates that the lattice undergoes a "nonthermal melting" process, where amorphization occurs with cold lattice. Our findings align with the conclusions of Daraszewicz *et al.*, and Medvedev *et al.* regarding nonthermal behaviors under different constraints [12, 13], suggesting that the nonthermal melting of laser-excited Au is primarily due to phonon softening induced by electron pressure relaxation process. We emphasize that under moderate non-equilibrium states ($T_\mathrm{e} \leq 2$ eV), electron pressure relaxation serves as the primary driving force for structural transformation in such noble metals with FCC structure.

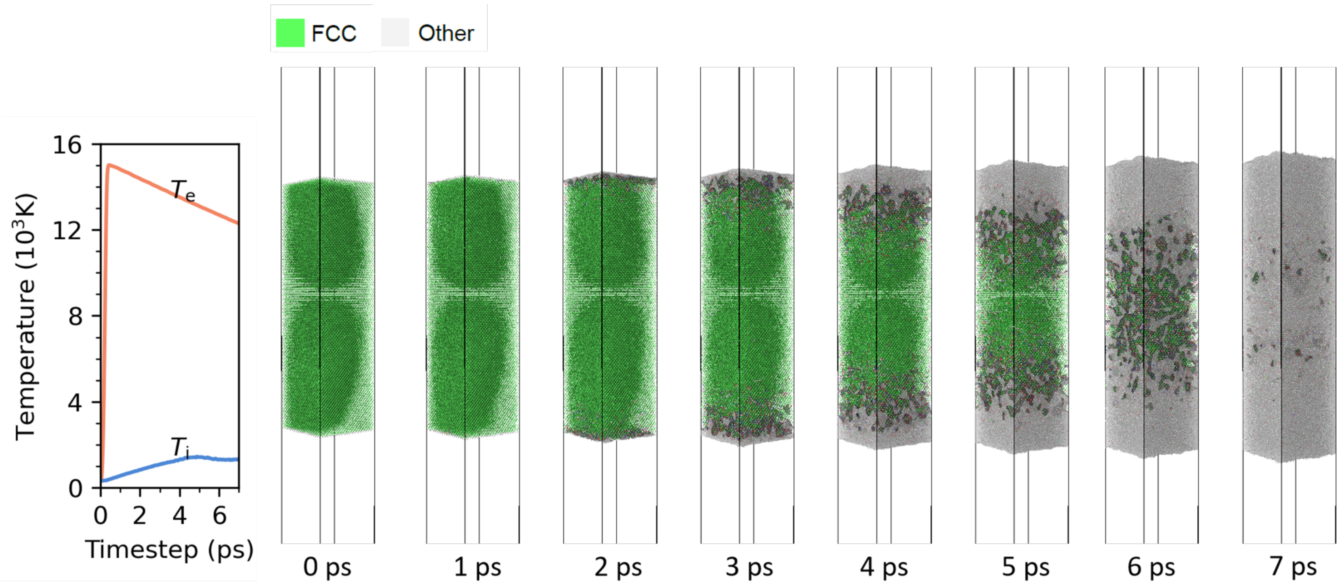

Supplementary Figure 12: Atomic configurations of laser-heated 35-nm-thick Au nanofilm under laser energy density of 0.80 MJ/kg, where the local symmetry of atoms is identified via PTM method. The green and grey color denotes FCC and disordered structure respectively. The atomic configurations are visualized by OVITO software [3].

Further, we performed large-scale TTM-DPMD simulations to capture the real response of a 35-nm-thick free-standing Au nanofilm (containing 13,7600 atoms) upon laser heating. Similar to laser-excited W, electronic pressure relaxation dynamics is also observed in laser-excited Au. Supplementary Fig.12 illustrates the ultrafast heterogeneous melting process in Au nanofilm, where the solid-liquid interface appeared from the free surface can be clearly identified. When the laser energy density is set to 0.80 MJ kg^{-1}, the initial maximum electron temperature is approximately $\sim 15,000$ K, yielding an electronic pressure contribution of ~ 20 GPa. With the release of electronic pressure, the surface atoms directly transforms into a disordered structure due to significant reduction in melting point. The melt front then moves inward at a speed of $2,916$ m s^{-1}, completing the melting process within 8 ps. These findings underscore the universal existence of electronic pressure, relaxation dynamics, and ultrafast heterogeneous melting in laser-excited metals.

[1] T. Tadano, Y. Gohda, and S. Tsuneyuki, J. Phys.-Condes. Matter **26**, 225402 (2014).
[2] P. M. Larsen, S. Schmidt, and J. Schi{o}tz, Modelling Simul. Mater. Sci. Eng. **24**, 055007 (2016).
[3] A. Stukowski, Modelling Simul. Mater. Sci. Eng. **18**, 015012 (2009).
[4] S. P. Coleman, D. E. Spearot, and L. Capolungo, Model. Simul. Mater. Sci. Eng. **21**, 055020 (2013).

[5] S. L. Daraszewicz, Y. Giret, H. Tanimura, D. M. Duffy, A. L. Shluger, and K. Tanimura, Appl. Phys. Lett **105**, 023112 (2015).
[6] M. Mo, S. Murphy, Z. Chen, P. Fossati, R. Li, Y. Wang, X. Wang, and S. Glenzer, Sci. Adv. **5**, eaaw0392 (2019).
[7] Z. Lin, L. V. Zhigilei, and V. Celli, Phys. Rev. B **77**, 075133 (2008).
[8] N. Medvedev and I. Milov, Phys. Rev. B **102**, 064302 (2020).
[9] N. A. Smirnov, Phys. Rev. B **111**, 014107 (2025).
[10] N. A. Smirnov, Phys. Rev. B **101**, 094103 (2020).
[11] E. Bévillon, J. P. Colombier, V. Recoules, and R. Stoian, Phys. Rev. B **89**, 115117 (2014).
[12] S. L. Daraszewicz, Y. Giret, N. Naruse, Y. Murooka, J. Yang, D. M. Duffy, A. L. Shluger, and K. Tanimura, Phys. Rev. B **88**, 184101 (2013).
[13] N. Medvedev and I. Milov, Sci. Rep. **10**, 12775 (2020).
